# Lung Metastases in Newly Diagnosed Esophageal Cancer: A Population-Based Study

**DOI:** 10.3389/fonc.2021.603953

**Published:** 2021-02-25

**Authors:** Jida Guo, Shengqiang Zhang, Huawei Li, Mohamed Osman Omar Hassan, Tong Lu, Jiaying Zhao, Linyou Zhang

**Affiliations:** Department of Thoracic Surgery, The Second Affiliated Hospital of Harbin Medical University, Harbin, China

**Keywords:** esophageal cancer, lung metastases, survival, treatment, SEER program

## Abstract

**Background:**

Esophageal cancer is one of the most common cancer types, with its most common distant metastatic site being the lung. Currently, population-based data regarding the proportion and prognosis of patients with esophageal cancer with lung metastases (ECLM) at the time of diagnosis is insufficient. Therefore, we aimed to determine the proportion of patients with ECLM at diagnosis, as well as to investigate the prognostic parameters of ECLM.

**Methods:**

This population-based observational study obtained data from the Surveillance, Epidemiology, and End Results (SEER) database registered between 2010 and 2016. Multivariable logistic regression was performed to identify predictors of the presence of ECLM at diagnosis. Multivariable Cox regression and competing risk analysis were used to assess prognostic factors in patients with ECLM. Median survival was estimated using Kaplan–Meier curves.

**Results:**

Of 10,965 patients diagnosed with esophageal cancer between 2010 and 2016, 713 (6.50%) presented with initial lung metastasis at diagnosis. Lung metastasis represented 27.15% of all cases with metastatic disease to any distant site. Considering all patients with esophageal cancer, multivariable logistic regression indicated that pathology grade, pathology type, T staging, N staging, race, and number of extrapulmonary metastatic sites were predictive factors for the occurrence of lung metastases at diagnosis. The median survival time of patients with ECLM was 4.0 months. Patients receiving chemotherapy or chemoradiotherapy had the longest median overall survival, 7.0 months. Multivariable Cox regression indicated that age, histology type, T2 staging, number of extrapulmonary metastatic sites, and treatment (chemotherapy, radiotherapy, or chemoradiotherapy) were independent predictors for overall survival (OS). Multivariable competing risk analysis determined that age, number of extrapulmonary metastatic sites, and treatment were independent predictors for esophageal cancer-specific survival (CSS).

**Conclusion:**

The findings of this study may provide important information for the early diagnosis of ECLM, as well as aid physicians in choosing appropriate treatment regimens for these patients.

## Introduction

Esophageal cancer is the seventh most prevalent malignant tumor and has the sixth highest mortality rate worldwide (1). In 2019, 18,440 new esophageal cancer cases and 16,171 esophageal cancer-related deaths were registered in the United States (2). In Western countries, the incidence of esophageal cancer—especially esophageal adenocarcinoma—has increased over the recent decades (3–5). At the time of diagnosis, approximately 50% of patients with esophageal cancer have metastases to distant lymph nodes or other organs (6, 7). In general, the most common distant metastasis organs for esophageal cancer are, in descending order, the liver, lung, bone, and brain (8–10). The prognosis of esophageal cancer patients with distant metastases is very poor, with a 5-year survival rate <5% (11, 12). In recent years, the overall 5-year survival rate for patients with metastatic esophageal cancer may increase to approximately 20% in many countries owing to the development of new treatment methods and use of targeted drugs (13). Nonetheless, the establishment of an optimal treatment for esophageal cancer with distant metastasis (M1) requires further studies and clinical trials.

Wu et al. (13) suggested that surgery combined with radiotherapy could improve survival in patients with metastatic esophageal cancer; however, other studies had different perspectives (14, 15). Tanaka et al. (11) proposed chemoradiotherapy as an effective treatment for esophageal squamous cell carcinoma (ESCC) patients with distant metastasis. Additionally, the National Comprehensive Cancer Network (NCCN) guidelines recommend solely supportive and palliative care for these patients (16). Thus, treatment strategies for patients with M1 esophageal cancer remain controversial. To the best of our knowledge, a few population-based studies on esophageal cancer with distant metastasis have been published (17, 18). However, these studies did not provide population-level estimates of prognosis parameters for patients newly diagnosed with esophageal cancer with lung metastases (ECLM). Therefore, a population-based study providing detailed information about ECLM remains necessary to clarify the epidemiologic characteristics and prognosis associated with this disease.

The NCCN guidelines recommend using computed tomography (CT) and positron emission tomography (PET)/CT to estimate the clinical stage of esophageal cancer and determine whether distant metastases are present (16). For lung metastasis diagnosis, CT may be combined with fiberoptic bronchoscopy, endoscopic ultrasonography, and chest X-ray (19). Although CT is a routine imaging technique to monitor potential lung metastasis, its detection at the earliest stage remains a great challenge because of its small size and low density (20). With the advent of multidetector computed tomography (MDCT), especially 64-slice systems, the detection of small pulmonary nodules has improved (21). PET/CT has great advantages for excluding metastatic lesions; moreover, its positive and negative predictive values for distant metastatic diseases reached 68 and 99%, respectively (22). However, PET/CT is expensive, making it not cost-effective to use in diagnosis and evaluation. Therefore, identifying esophageal cancer patients who are at high-risk of lung metastases and determining predictive factors for ECLM occurrence are important. We believe that combining predictive factors with 64-slice MDCT may improve early and accurate diagnosis of ECLM.

In this population-based study, we used data of esophageal cancer patients with or without lung metastases from the Surveillance, Epidemiology, and End Results (SEER) database between 2010 and 2016 to determine the incidence of lung metastasis at diagnosis and to investigate predictive factors of lung metastasis detection at diagnosis on a population level. Additionally, we analyzed prognostic factors affecting the survival of ECLM patients and compared the effect of different treatments in disease prognosis.

## Methods

### Cohort Selection and Data Collection

This retrospective study analyzed publicly available data from the SEER database. We used SEER*stat software version 8.3.4 (with additional treatment from 1975 to 2016) to extract data from the SEER-18 database, which includes information on cancer incidence, treatment, and survival for approximately 28% of the US population (23). The most recent datapoints available in the SEER database date from 2016 based on submissions up until November 2018; these became available in April 2019. Moreover, the SEER database did not include any information regarding metastases location until 2010. Therefore, we analyzed data from patients diagnosed with esophageal cancer between January 1, 2010 and December 31, 2016, including as many medical records as possible.

**Figure 1:**
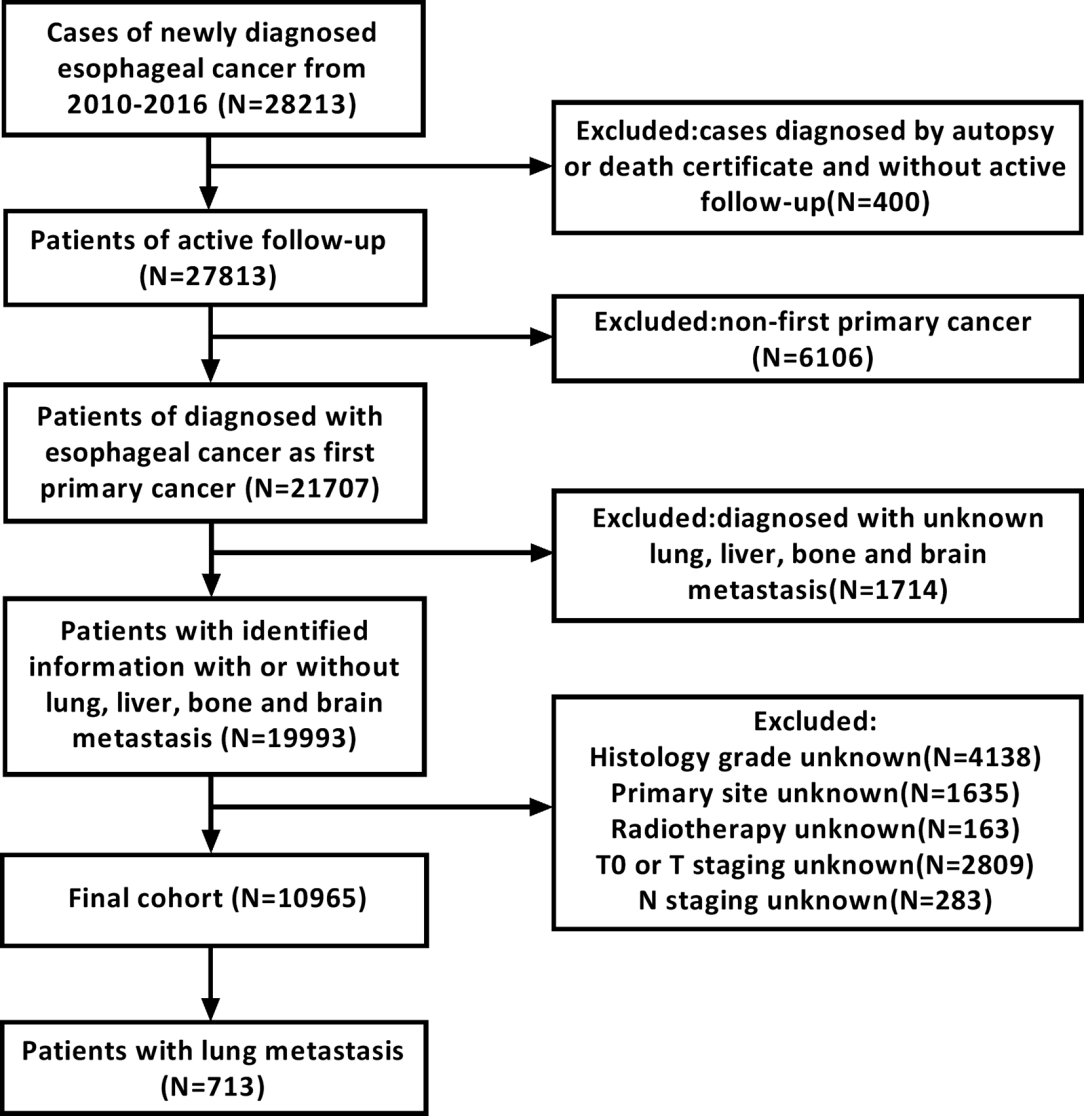
Flowchart of the esophageal cancer patients selection from SEER database.

We screened the records of 28,213 patients who were initially diagnosed with esophageal cancer between 2010 and 2016. All patients were 18 years old or older. Exclusion criteria were as follows: 1) patients diagnosed based on death certificate or autopsy; 2) patients without active follow-up; 3) patients whose esophageal cancer was not the primary cancer; 4) lack of information about lung, liver, bone, and brain metastases at diagnosis; 5) patients whose esophageal cancer was stage T0; and 6) lack of information regarding histology grade, primary site, radiotherapy regimen, T staging, and N staging. A detailed data extraction flowchart is shown in **Figure 1**. Finally, 10,965 patients were included in the final study cohort. Of those, 2,626 patients were diagnosed with metastases to any distant site and 713 patients were diagnosed with lung metastases. Subsequently, we collected clinical and sociodemographic variables to conduct a descriptive statistical analysis and summarize the demographic and tumor characteristics of patients. Clinical variables included sex, age at diagnosis, tumor location, pathology grade, pathology type, tumor staging, T staging, N staging, treatment, race, and extra lung metastases number. Sociodemographic variables included insurance status, marital status, high school education, and median family income.

**Figure 2:**
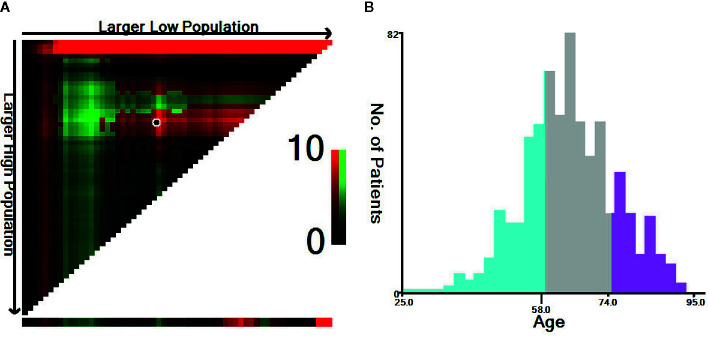
Identification of the optimal age cut-off points for esophageal cancer patients with lung metastasis. **(A)** X-tile plots based on age according to esophageal cancer-specific mortality. The plots show χ^2^ log-rank values; the brightest pixel represents the maximum χ^2^ log-rank value. **(B)** Distribution of patients according to age ranging from 25 to 95 years old. The optimal age cut-off of ECLM patients age is shown as 58 and 74 years old (χ^2^ = 9.65, *P* < 0.001).

In this study, the optimal age cut-off points were determined using the X-tile program (http://www.tissuearray.org/rimmlab/), which can divide the cohort into three subsets and determine two optimal cut-off values using the minimum *p-*value from the log-rank χ^2^ statistics for patients’ age based on survival rates (24). Therefore, we used X-tile to identify the optimal age cut-off based on the esophageal cancer-specific mortality rate of ECLM patients. As shown in **Figure 2**, the optimal age cut-off points were 58 and 74 years; thus, we stratified the cohort into three age groups: 18–58 years old, 59–74 years old, and ≥75 years old. Tumor locations included lesions in the upper, middle, and lower esophagus, as well as overlapping lesions. Cancer pathology grade was classified into three categories: well-differentiated (Grade I), moderately differentiated (Grade II), and poorly differentiated or undifferentiated (Grade III/IV). Histological types were determined using the International Classification of Diseases for Oncology, third edition (ICD-O-3) (adenocarcinoma: 8140, 8144, 8145, 8210, 8211, 8244, 8255, 8260–8263, and 8323; squamous carcinoma: 8051, 8052, 8070–8075, and 8083; others: 8000, 8010, 8013, 8020, 8033, 8041, 8046, 8094, 8480, 8490, 8560, 8574, and 8980).

TNM staging was based on the seventh edition of the Cancer Staging Manual of the American Joint Committee on Cancer (AJCC) (25). Because the eighth edition is the most commonly used classification at present, we converted the codes from the previous edition to their corresponding eighth-edition codes. T staging included T1, T2, T3, and T4; N staging included N0, N1, N2, and N3. Patients were also stratified according to treatment regimens: those who received radiotherapy (Ra), those who received chemotherapy (Che), those who received chemoradiotherapy (Che+Ra), and those who received none of these (No). In this study, the number and proportion of patients who received chemoradiotherapy were determined based on with/without chemotherapy patients number and with/without radiotherapy patients number.

Race contained white, black, and others. Insurance status was classified into insured, uninsured, and unknown. Marital status was divided into married, unmarried, and unknown. Educational levels were stipulated based on 10% increments with high school education as the base level. Median household income levels were defined using $20,000 increments. High school education and median family income were derived from the US Census American Community Survey obtained from the SEER*Stat software (26). Additionally, we investigated if the number of extrapulmonary metastases (in the liver, bone, brain, and other sites) at diagnosis was associated with the presence of metastasis in the lung and prognosis of ECLM patients.

### Statistical Analysis

We used descriptive statistics to calculate the absolute number, percentage, and median survival (in months) of patients with ECLM at diagnosis. ECLM incidence was determined based on the proportion of ECLM patients among the entire cohort, as well as among those diagnosed with metastatic esophageal cancer to any distant site. All data were stratified by sex, age, tumor location, and other variables. Multivariable logistic regression was used to determine predictive factors for the presence of lung metastases at diagnosis. The variables included in the multivariable logistic regression analysis were selected based on an univariable logistic regression analysis to identify statistically significant variables (*P* < 0.05).

Survival estimates were obtained using the Kaplan–Meier method and compared using the log-rank test. We conducted an univariable Cox regression model to identify statistically significant variables (*P* < 0.05); subsequently, these variables were included in the multivariable Cox regression model to determine the covariates associated with all-cause mortality. To analyze the variables that affected esophageal cancer-specific mortality, we conducted Fine and Gray’s competing risk regression for univariable and multivariable analysis (27).

Statistical analyses were performed using SPSS (version 25.0; IBM Corp., Armonk, NY, USA) and R (version 3.3.2; R Foundation for Statistical Computing, Vienna, Austria) software. Logistic and Cox regression analyses were performed using SPSS. Prism 7.0 (GraphPad Software, San Diego, CA, USA) was used to plot Kaplan–Meier survival curves. R was used for competing risk analysis using the package “cmprsk” (version 2.2-72014). Two-sided *P*-values <0.05 were considered statistically significant.

## Results

### Patient Characteristics

Overall, 10,965 patients were diagnosed with esophageal cancer between 2010 and 2016 in the USA. Of these, 8,867 (80.87%) were men and 2,098 (19.13%) were women, with a median age of 66 years (range, 18–102 years). The proportion of patients diagnosed with esophageal adenocarcinoma was more than two-fold of patients diagnosed with ESCC (64.30 *vs*. 28.15%). Patients’ median survival was 11 months. Among all patients diagnosed with esophageal cancer, 2,626 presented with metastatic disease to any distant site and 713 presented with metastases in the lung. Regarding those with lung metastases, 594 (83.31%) were men and 119 (16.69%) were women. Moreover, those with lung metastases represented 6.50% of the entire cohort and 27.15% of patients with metastatic disease to any distant site. Patients’ clinical and demographic characteristics are shown in **Table 1**.

**Table 1:** Clinical Characteristics of Patients With Esophagus Cancer With Identified Lung Metastases at Diagnosis.

Variable	Patients, No.			Proportion of Lung Metastases, %		Survival Among Patients With Lung Metastases,Median (IQR), mo
	With Esophagus Cancer (n = 10,965)	With metastatic Disease (n = 2,626)	With Lung Metastases(n=713)	Among EntireCohort	Among Subset With Metastatic Disease	
Sex						
Male	8,867	2224	594	6.7	26.7	4.0 (1.0–9.0)
Female	2,098	402	119	5.7	29.6	5.0 (1.0–10.0)
Age at diagnosis, Y						
18–58	2,830	835	205	7.2	24.6	5.0 (2.0–11.0)
59–74	5,737	1,326	374	6.5	28.2	4.0 (1.0–9.3)
≥75	2,398	465	134	5.6	28.8	3.0 (1.0–7.3)
Year at diagnosis						
2010	1,553	378	98	6.3	25.9	4.5 (2.0–9.5)
2011	1,542	365	102	6.6	28.7	6.0 (2.0–11.0)
2012	1,546	383	112	7.2	29.2	3.0 (1.0–8.0)
2013	1,607	375	105	6.5	28.0	5.0 (1.5–11.5)
2014	1,596	363	93	5.8	25.6	5.0 (2.0–12.0)
2015	1,681	428	109	6.5	25.5	3.0 (1.0–8.5)
2016	1,440	343	94	6.5	27.4	3.0 (1.0–6.0)
Tumor location						
Upper	739	112	51	6.9	45.5	4.0 (2.0–10.0)
Middle	1,731	340	121	7.0	35.6	4.0 (2.0–10.0)
Lower	7,988	2,008	485	6.1	24.2	4.0 (1.0–9.0)
Overlapping	507	166	56	11.0	33.7	2.0 (1.0–7.75)
Pathology grade						
Grade I	730	69	18	2.5	26.1	5.0 (1.75–13.5)
Grade II	4,670	917	284	6.1	31.0	5.0 (2.0–10.0)
Grade III/IV	5,565	1,640	411	7.4	25.1	4.0 (1.0–9.0)
Histology type						
Adenocarcinoma	7,050	1,826	441	6.3	24.2	5.0 (2.0–10.0)
Squamous	3,087	568	226	7.3	39.8	4.0 (1.0–9.0)
Others^a^	828	232	46	5.6	19.8	1.5 (0.0–5.0)
Tumor staging^b^						
I	1,990	0	0	0	0	NA
II	2,453	0	0	0	0	NA
III	3,869	0	0	0	0	NA
IV	2,626	2,626	713	27.2	27.2	4.0 (1.0–9.0)
T staging^b^						
T1	3,071	846	254	8.3	30.0	3.0 (1.0–9.0)
T2	1,435	188	31	2.2	16.5	6.0 (1.0–9.0)
T3	4,984	866	185	3.7	21.4	6.0 (3.0–13.0)
T4	1,475	726	243	16.5	33.5	3.0 (1.0–7.0)
N staging^b^						
N0	5,107	584	175	3.4	30.0	3.0 (1.0–9.0)
N1	4,704	1471	413	8.8	28.1	4.0 (1.0–10.0)
N2	661	340	64	9.7	18.8	5.0 (2.0–9.0)
N3	493	231	61	12.4	26.4	3.0 (1.0–8.0)
Treatment^c^						
No	2,265	508	179	7.9	35.2	1.0 (0.0–2.0)
Ra	727	294	102	14.0	34.7	2.0 (1.0–5.0)
Che	1,303	854	204	15.7	23.7	7.0 (3.0–13.0)
Che+Ra	6,670	970	228	3.4	23.5	7.0 (4.0–12.0)
Race						
White	9,349	2,239	570	6.1	25.5	4.0 (1.0–9.0)
Black	995	235	94	9.4	40.0	3.0 (1.0–8.25)
Others^d^	586	142	47	8.0	33.1	5.0 (2.0–11.0)
Unknown	35	10	2	5.7	20.0	1.5 (0.0–NA)
Insurance status						
Insured	10,472	2,482	666	6.4	26.8	4.0 (1.0–9.25)
Uninsured	335	113	36	10.7	31.9	3.0 (1.0–5.75)
Unknown	158	31	11	7.0	35.5	3.0 (0.0–13.0)
Marital status						
Married	6,171	1,472	364	5.9	24.7	4.5 (2.0–10.0)
Unmarried	4,267	1,050	313	7.3	29.8	4.0 (1.0–9.0)
Unknown	527	104	36	6.8	34.6	5.5 (1.25–13.0)
High school education						
0–10%	3,235	753	191	5.9	25.4	5.0 (2.0–10.0)
10–20%	5,640	1,381	378	6.7	27.4	4.0 (1.0–9.0)
20–30%	1,950	426	137	7.0	29.7	4.0 (1.0–9.0)
30–40%	140	30	7	5.0	23.3	3.0 (3.0–13.0)
Median household income						
20,000–40,000	105	17	4	3.8	23.5	2.0 (0.25–3.0)
40,000–60,000	2,291	574	166	7.2	28.9	4.0 (1.0–9.0)
60,000–80,000	4,256	1,041	283	6.6	27.2	4.0 (1.0–9.0)
80,000–100,000	2,821	636	160	5.7	25.2	5.0 (1.0–11.0)
>100,000	1,492	358	100	6.7	27.9	5.0 (2.0–9.75)
Extrapulmonary metastatic sites to liver, bone, brain, and others No.						
0	9,250	911	293	3.2	32.1	5.0 (2.0–11.0)
1	1,353	1,353	294	21.7	21.7	4.0 (1.0–9.0)
2	316	316	98	31.0	31.0	3.0 (1.0–6.0)
3	46	46	28	60.9	60.9	2.0 (2.0–6.0)

IQR, interquartile range, CI, confidence interval;

a Including signet ring cell carcinoma, Mucinous carcinoma, etc.

b According to the eighth edition of the AJCC Cancer Staging manual.

c Including No, Without Radiotherapy or Chemotherapy; RA, Radiotherapy; Che, Chemotherapy; Ra+Che, Radiotherapy plus Chemotherapy.

d Including Hispanic, Asian, etc.

### Predictors for the Presence of Lung Metastases

Univariable logistic regression analysis (**Supplementary Table 1**) identified 10 statistically significant variables (*P* < 0.05) among the entire cohort: age, tumor location, pathology grade, pathology type, T staging, N staging, race, insurance status, marital status, and number of extrapulmonary metastatic sites. These variables were included in the multivariable logistic regression analysis. As shown in **Table 2**, pathology grade, pathology type, T staging, N staging, race, and number of extrapulmonary metastatic sites were identified as statistically significant among the entire cohort.

**Table 2:** Multivariable Logistic Regression for the Presence of Lung Metastases at Diagnosis of Esophagus Cancer.

Variable	Patients, No		Among Entire Cohort	
	Patients (n = 10,965)	With Lung Metastases (n = 713)	OR (95% CI)	*P* Value
Sex				
Male	8,867	594	NA	NA
Female	2,098	119	NA	NA
Age at diagnosis (Year)				
18–58	2,830	205	1 (reference)	NA
59–74	5,737	374	1.131 (0.930–1.375)	0.218
≥75	2,398	134	1.142 (0.891–1.465)	0.293
Tumor location				
Upper	739	51	1 (reference)	NA
Middle	1,731	121	1.003 (0.694–1.450)	0.986
Lower	7,988	485	0.880 (0.614–1.261)	0.485
Overlapping	507	56	1.295 (0.826–2.032)	0.260
Pathology grade				
Grade I	730	18	1 (reference)	NA
Grade II	4,670	284	2.031 (1.222–3.375)	0.006
Grade III/IV	5,565	411	1.975 (1.190–3.276)	0.008
Histology type				
Adenocarcinoma	7,050	441	1 (reference)	NA
Squamous	3,087	226	1.349 (1.064–1.710)	0.013
Others^a^	828	46	0.831 (0.591–1.168)	0.286
T staging^b^				
T1	3,071	254	1 (reference)	NA
T2	1,435	31	0.297 (0.188–0.414)	<0.001
T3	4,984	185	0.441 (0.355–0.547)	<0.001
T4	1,475	243	1.287 (1.040–1.592)	0.020
N staging^b^				
N0	5,107	175	1 (reference)	NA
N1	4,704	413	2.055 (1.682–2.512)	<0.001
N2	661	64	2.076 (1.482–2.909)	<0.001
N3	493	61	2.419 (1.695–3.452	<0.001
Race				
White	9,394	570	1 (reference)	NA
Black	995	94	1.359 (1.028–1.797)	0.031
Others^c^	586	47	1.293 (0.912–1.832)	0.149
Unknown	35	2	0.768 (0.166–3.546)	0.735
Insurance status				
Insured	10,472	666	1 (reference)	NA
Uninsured	335	36	1.120 (0.749–1.674)	0.581
Unknown	158	11	1.090 (0.557–2.134)	0.802
Marital status				
Married	6,171	364	1 (reference)	NA
Unmarried	4,267	313	1.161 (0.973–1.384)	0.097
Unknown	527	36	1.455 (0.987–2.143)	0.058
High school education (per 10% increase)	10,965	713	NA	NA
Median household income (per 20,000 increase)	10,965	713	NA	NA
Extrapulmonary metastatic sites to liver, bone, brain, and others No.			
0	9,250	293	1 (reference)	NA
1	1,353	294	6.294 (5.194–7.627)	<0.001
2	316	98	10.187 (7.643–13.576)	<0.001
3	46	28	32.767 (17.187–62.472)	<0.001

CI, confidence interval; OR, odds ratio

a Including signet ring cell carcinoma, Mucinous carcinoma, etc.

b According to the eighth edition of the AJCC Cancer Staging manual.

c Including Hispanic, Asian, etc.

Specifically, the following factors were associated with greater odds of lung metastasis presence at diagnosis: pathology grade II (*vs.* grade I; odds ratio [OR], 2.031; 95% confidence interval [CI], 1.222–3.375; *P =* 0.006), pathology grade III/IV (*vs.* grade I; OR, 1.975; 95% CI, 1.190–3.276; *P* = 0.008), squamous cell carcinoma (*vs.* adenocarcinoma; OR, 1.349; 95% CI, 1.064–1.710; *P* = 0.013), stage T4 (*vs.* T1; OR, 1.287; 95% CI, 1.040–1.592; *P* = 0.020), stage N1 (*vs.* N0; OR, 2.055; 95% CI, 1.682–2.512; *P* < 0.001), stage N2 (*vs.* N0; OR, 2.076; 95% CI, 1.482–2.909; *P* < 0.001), stage N3 (*vs.* N0; OR, 2.419; 95% CI, 1.695–3.452; P < 0.001), black race (*vs.* white; OR, 1.359; 95% CI, 1.028–1.797; *P* = 0.031), 1 extrapulmonary metastatic site (*vs.* 0 extrapulmonary metastatic site; OR, 6.294; 95% CI, 5.194–7.627; *P* < 0.001), 2 extrapulmonary metastatic sites (*vs.* 0 extrapulmonary metastatic site; OR, 10.187; 95% CI, 7.643–13.576; *P* < 0.001), and 3 extrapulmonary metastatic sites (*vs.* 0 extrapulmonary metastatic site; OR, 32.767; 95% CI, 17.187–62.472; *P* < 0.001). Conversely, the multivariable model indicated that sex, age, tumor location, insurance status, and marital status were not associated with the risk of lung metastasis presence at diagnosis. Additionally, stage T2 (*vs.* T1; OR, 0.297; 95% CI, 0.188–0.414; *P* < 0.001) and stage T3 (*vs.* T1; OR, 0.441; 95% CI, 0.355–0.547; *P* < 0.001) were associated with marginally lower odds of lung metastasis presence at diagnosis.

Considering these results, patients with esophageal cancer who presented with poor tumor grade, squamous cell carcinoma, T4 staging, late N staging, and presence of more metastatic sites, as well as black patients, had a higher risk of presenting with lung metastases at diagnosis, whereas T2 and T3 staging were considered protective factors for lung metastasis at diagnosis.

### Survival

For all-cause mortality among patients with metastatic disease to any distant site, univariable Cox regression analysis identified twelve variables that were significantly associated with overall survival (*P* < 0.05): sex, age, pathology type, T staging, N staging, number of extrapulmonary metastatic sites, race, insurance situation, marital status, high school education, median household income, and treatment (**Supplementary Table 2**). These variables were included in the multivariable Cox regression analyses. The statistics showed that eight variables were significantly associated with all-cause mortality for esophageal cancer patients with distant metastases (*P* < 0.05), including sex, age, pathology type, T staging, number of extrapulmonary metastatic sites, marital status, median household income, and treatment. Detailed statistical results are shown in **Supplementary Table 3**.

Univariable Cox regression analysis for all-cause mortality among patients with ECLM identified seven variables that were significantly associated with overall survival (*P* < 0.05): age, tumor location, pathology type, T staging, number of extrapulmonary metastatic sites, median household income, and treatment type. Regarding esophageal cancer-specific mortality, univariable competing risk analysis identified four variables that were significantly associated with cancer-specific survival (*P* < 0.05): age, pathology type, number of extrapulmonary metastatic sites, and treatment type. Details of the univariable analysis of mortality among patients with ECLM are displayed in **Supplementary Table 4**.

The variables identified using univariable analyses were included in multivariable analyses (**Table 3**). Multivariable Cox regression analysis indicated that the following factors were associated with an increase in all-cause mortality among patients with ECLM: age ≥75 years (*vs.* 18–58 years; hazard ratio [HR], 1.481; 95% CI, 1.168–1.877; *P* = 0.001), other pathology types (*vs.* adenocarcinoma; HR, 1.769; 95% CI, 1.275–2.454; *P* = 0.001), 1 extrapulmonary metastatic site (*vs.* 0 extrapulmonary metastatic site; HR, 1.190; 95% CI, 1.333–1.107; *P*=0.002), and ≥2 extrapulmonary metastatic sites (*vs.* 0 extrapulmonary metastatic site; HR, 1.822; 95% CI, 1.419–2.339; *P* < 0.001). Conversely, stage T2 (*vs.* T1; HR, 0.633; 95% CI, 0.404–0.992; *P* = 0.046), Ra treatment (*vs.* No treatment; HR, 0.648; 95% CI, 0.500–0.839; *P* = 0.001), Che treatment (*vs.* No treatment; HR, 0.260; 95% CI, 0.209–0.325; *P* < 0.001), and Che+Ra treatment (*vs.* No treatment; HR, 0.259; 95% CI, 0.206–0.325; *P* < 0.001) were significantly associated with a decrease in all-cause mortality. Tumor location and median household income were not significantly associated with all-cause mortality (*P* > 0.05).

**Table 3:** Multivariable Cox Regression for All-Cause Mortality and Esophageal Cancer Specific Mortality Among Patients With Lung Metastases.

Variable	Patients, No.		All-cause mortality		Cancer-specific mortality	
	Patients (n = 10,965)	With Lung Metastases(n = 713)	Hazard Ratio (95% CI)	*P* Value	Hazard Ratio (95% CI)	*P* Value
Sex						
Male	8,867	594	NA	NA	NA	NA
Female	2,098	119	NA	NA	NA	NA
Age at diagnosis, Y						
18–58	2,830	205	1 (reference)	NA	1 (reference)	NA
59–74	5,737	374	1.147 (0.954–1.379)	0.144	1.341 (0.771–2.334)	0.300
≥75	2,398	134	1.481 (1.168–1.877)	0.001	2.359 (1.346–4.135)	0.003
Tumor location						
Upper	739	51	1 (reference)	NA	NA	NA
Middle	1,731	121	1.159 (0.818–1.641)	0.407	NA	NA
Lower	7,988	485	1.117 (0.788–1.584)	0.502	NA	NA
Overlapping	507	56	1.385 (0.917–2.093)	0.121	NA	NA
Pathology grade						
Grade I	730	18	NA	NA	NA	NA
Grade II	4,670	284	NA	NA	NA	NA
Grade III/IV	5,565	411	NA	NA	NA	NA
Histology type						
Adenocarcinoma	7,050	441	1 (reference)	NA	1 (reference)	NA
Squamous	3,087	226	1.117 (0.905–1.378)	0.421	1.155 (0.745–1.739)	0.520
Others^a^	828	46	1.769 (1.275–2.454)	0.001	1.468 (0.760–2.907)	0.250
T staging^b^						
T1	3,071	254	1 (reference)	NA	NA	NA
T2	1,435	31	0.633 (0.404–0.992)	0.046	NA	NA
T3	4,984	185	0.864 (0.696–1.071)	0.183	NA	NA
T4	1,475	243	1.095 (0.911–1.318)	0.334	NA	NA
N staging^b^						
N0	5,107	175	NA	NA	NA	NA
N1	4,704	413	NA	NA	NA	NA
N2	661	64	NA	NA	NA	NA
N3	493	61	NA	NA	NA	NA
Extrapulmonary metastatic sites to liver, bone, brain, and others No.						
0	9,250	293	1 (reference)	NA	1 (reference)	NA
1	1,353	294	1.333 (1.107–1.605)	0.002	1.278 (1.026–1.593)	0.029
≥2	362	126	1.822 (1.419–2.339)	<0.001	1.469 (1.099–1.963)	0.009
Race						
White	9,394	570	NA	NA	NA	NA
Black	995	94	NA	NA	NA	NA
Others^c^	586	47	NA	NA	NA	NA
Unknown	35	2	NA	NA	NA	NA
Insurance status						
Insured	10,472	666	NA	NA	NA	NA
Uninsured	335	36	NA	NA	NA	NA
Unknown	158	11	NA	NA	NA	NA
Marital status						
Married	6,171	364	NA	NA	NA	NA
Unmarried	4,267	313	NA	NA	NA	NA
Unknown	527	36	NA	NA	NA	NA
High school education (per 10% increase)	10,965	713	NA	NA	NA	NA
Median household income (per 20000 increase)	10,965	713	0.958 (0.882–1.040)	0.307	NA	NA
Treatment^d^						
No	2,265	179	1 (reference)	NA	1 (reference)	NA
Ra	727	102	0.648 (0.500–0.839)	0.001	0.965 (0.620–1.500)	0.870
Che	1,303	204	0.260 (0.209–0.325)	<0.001	0.196 (0.098–0.391)	<0.001
Che+Ra	6,670	228	0.259 (0.206–0.325)	<0.001	0.223 (0.117–0.423)	<0.001

CI, confidence interval

^a^Including signet ring cell carcinoma, Mucinous carcinoma, etc.

^b^According to the eighth edition of the AJCC Cancer Staging manual.

^c^Including Hispanic, Asian, etc.

^d^Including No, Without Radiotherapy or Chemotherapy; RA, Radiotherapy; Che, Chemotherapy; Ra+Che, Radiotherapy plus Chemotherapy.

Multivariable competing risk analysis for esophageal cancer-specific mortality among patients with ECLM identified the following factors associated with an increase in esophageal cancer-specific mortality: age ≥75 years (*vs.* 18–58 years; HR, 2.359; 95% CI, 1.346–4.135; *P* = 0.003), 1 extrapulmonary metastatic site (*vs.* 0 extra-pulmonary metastatic site; HR, 1.278; 95% CI, 1.026–1.593; *P*=0.029), ≥2 extrapulmonary metastatic sites (*vs.* 0 extrapulmonary metastatic site; HR, 1.469; 95% CI, 1.099–1.963; *P* = 0.009). Conversely, Che treatment (*vs.* No treatment; HR, 0.169; 95% CI, 0.098–0.391; *P* < 0.001) and Che+Ra treatment (*vs.* No treatment; HR, 0.223; 95% CI, 0.117–0.423; *P* < 0.001) were significantly associated with a decrease in esophageal cancer-specific mortality. Pathology type (including squamous cell carcinoma, adenocarcinoma, and others) and Ra treatment were not associated with esophageal cancer-specific mortality.

Multivariable Cox regression analysis indicated that other histology types were associated with poor overall survival, whereas T2 staging was associated with improved overall survival. Furthermore, considering the results of both multivariable models, patients with ECLM who were 75 years old or older had a worse prognosis, which was consistent with the optimal age cut-off points for patients with ECLM (**Figure 2**). Moreover, the multivariable analyses indicated that those with an extensive systemic disease at diagnosis had poor survival, while those who received positive treatment (radiotherapy, chemotherapy, or chemoradiotherapy) had improved survival.

**Figure 3:**
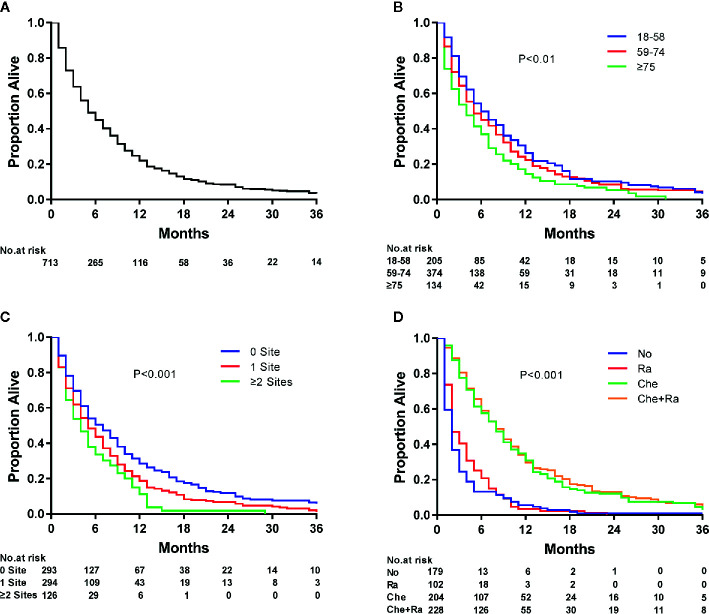
Kaplan–Meier analysis of overall survival among esophageal cancer patients with lung metastasis at diagnosis. **(A)** overall, **(B)** stratified by age, **(C)** stratified by the extent of extrapulmonary metastatic disease, and **(D)** stratified by type of treatment.

Survival estimates obtained using the Kaplan–Meier method indicated that the median survival of patients with ECLM was 4.0 months (interquartile range [IQR], 1.0–9.0 months) (**Figure 3A**). The median survival of patients with ECLM aged 18–58 years was 5.0 months (IQR, 2.0–11.0 months); 59–74 years, 4.0 months (IQR, 1.0–9.3 months); and ≥75 years, 3.0 months (IQR, 1.0–7.3 months) (**Figure 3B**). The median survival of those with no extrapulmonary metastatic site was 5.0 months (IQR, 2.0–11.0 months); 1 extrapulmonary metastatic site, 4.0 months (IQR, 1.0–9.0 months); with ≥2 extrapulmonary metastatic sites, 3.0 months (IQR, 1.0–6.0 months) (**Figure 3C**). Finally, the median survival of patients with ECLM who received No treatment was 1.0 month (IQR, 0.0–2.0 months); Ra treatment, 2.0 months (IQR, 1.0–5.0 months); Che treatment, 7.0 months (IQR, 3.0–13.0 months); and Che+Ra treatment, 7.0 months (IQR, 4.0–12.0 months) (**Figure 3D**). In summary, those treated with chemotherapy or chemoradiotherapy had the best prognosis, while those who received no treatment had the worst prognosis.

Among all patients with ECLM, 102 (14.31%) received radiotherapy, 204 (28.61%) received chemotherapy, and 228 (31.98%) received chemoradiotherapy. Among the entire cohort, 727 (6.63%) patients received radiotherapy, 1,303 (11.88%) received chemotherapy, and 6,670 (60.83%) received chemoradiotherapy. Therefore, those who received chemoradiotherapy represented the majority in the entire cohort and among patients with ECLM.

## Discussion

To the best of our knowledge, this is the first population-based study to describe the incidence proportion of ECLM and analyze the predictive and prognostic factors associated with the outcomes of patients with ECLM at diagnosis. We used the most recent data on esophageal cancer available in the SEER database, identifying and including 713 patients with ECLM in our study—possibly the largest number of patients with ECLM included in a study. We used descriptive statistical and logistic regression analyses to investigate the factors associated with ECLM at diagnosis. Moreover, we used three statistical methods (Kaplan–Meier curves, Cox regression, and competing risk analyses) to obtain survival estimates. The results presented in this study may aid physicians in timely detecting lung metastases and choosing appropriate treatment methods.

We demonstrated that 6.50% of patients with esophageal cancer had lung metastases at diagnosis, and 27.15% of esophageal cancer patients with any distant metastases at diagnosis had lung metastases. These results were similar to those of a previous study that also used data from the SEER database (17); conversely, our results were higher than those reported by another study based on the SEER database (28). This discrepancy may be caused by the use of different exclusion criteria. Nonetheless, our study included a larger cohort than the abovementioned studies, indicating that our analysis had greater statistical power. A previous study reported that lung metastases represented 20% of all metastatic esophageal cancer cases (14), a proportion slightly lower than that found in our study. This difference may be explained by an increase in the proportion of metastatic diseases over the past 20 years, as well as the development of diagnostic technologies that can detect the presence of lung metastases more efficiently. An autopsy-based study reported that 52% of patients had lung metastases, the largest proportion ever reported (29). The authors of this study hypothesized that differences in race and sex between patients included in their cohort and those included in other studies were the main reason for this discrepancy, as well as the use of data with very few postoperative deaths.

Our and other studies have shown that patients with squamous cell carcinoma were more likely to develop lung metastasis compared with those with other esophageal cancer histological types (17, 28). The aforementioned autopsy-based study (29) was based solely on autopsy findings of patients with ESCC, representing an additional reason for the high proportion of lung metastases found in their study. Another autopsy-based study reported that 31% of patients with metastatic esophageal cancer had lung metastases (30), a proportion slightly higher than that found in our study. Their autopsy series included patients with recurred or post-treatment lung metastases, whereas we exclusively included those with lung metastases at diagnosis. In addition, metastases are more easily detected during autopsy than during physical and imaging examinations performed at the time of esophageal cancer diagnosis. Accordingly, the proportion of patients with ECLM found in our study and in other SEER-based studies are consistently lower than those found in autopsy-based studies (17, 28–30).

In our study, we used multivariable logistic regression analysis to determine predictive factors for the presence of lung metastasis at diagnosis and identify those at increased risk of having lung metastases. We showed that patients with poor tumor grade, squamous cell carcinoma histological type, T4 staging, late N staging, and more extrapulmonary metastatic sites, as well as black patients, had an increased risk of having lung metastases at diagnosis compared with the entire cohort. Regarding pathology grades, among the entire cohort, 6.66, 42.59, and 50.75% of patients with lung metastases had diseases grade I, II, and III/IV, respectively. Among those with metastatic esophageal cancer, 2.63, 34.92, and 62.45% of patients with lung metastases had diseases grade I, II, and III/IV, respectively. Those with disease grade II and III/IV had a significantly greater likelihood of presenting with lung metastases at diagnosis than those with disease grade I. These results are in accordance with previous studies (17, 31, 32). Higher pathology grades are considered more malignant; accordingly, several studies have demonstrated that pathology grade is a strong survival predictor, with higher grades indicating a poor prognosis (33, 34). Nonetheless, the mechanism behind the association between higher pathology grades and lung metastasis occurrence requires further elucidation.

Regarding tumor pathology types, patients diagnosed with squamous cell carcinoma had a higher proportion of lung metastases than those diagnosed with adenocarcinoma. This finding is in accordance with previously mentioned studies (17, 28), as well as with other Japanese studies that reported the lung as the most common distant metastasis site in ESCC (35, 36). We hypothesized that the higher proportion of lung metastasis in ESCC may be related with tumor location because most esophageal adenocarcinomas were located in the lower esophagus, whereas ESCC was more evenly distributed throughout the upper, middle, and lower esophagus, with the middle esophagus representing the largest proportion (8, 31). However, the underlying mechanisms of distant metastasis responsible for the differences observed between these two pathology subtypes remain unclear.

Regarding T staging, tumors in T4 stage had a significantly higher percentage of lung metastasis compared with those of tumors in T2 and T3 stages. Furthermore, tumors in late N staging had a higher proportion of lung metastasis than that of tumors in early N staging. Accordingly, a previous study reported that T and N staging were the greatest contributors in metastasis prediction (32). Sakanaka et al. (37) demonstrated that patients with larger metastatic lymph nodes were at a higher risk of developing diseases with distant metastases. Therefore, many studies have recognized late T staging and N staging as important factors for the occurrence of distant metastasis in esophageal cancer. In our study, T2 and T3 staging were associated with a lower risk of lung metastasis at diagnosis. Most T and N staging included in the SEER database were based on clinical staging using CT and other imaging examinations; consequently, the staging may be less accurate than pathological and autopsy-based staging used in other studies (38).

Black patients had a significantly greater likelihood of presenting with lung metastases at diagnosis than white patients. The reason for this difference remains unknown and requires further elucidation (38). In our study, esophageal cancer patients with more extrapulmonary metastatic sites had a significantly higher risk of lung metastasis. This finding is similar to those of studies on other metastatic malignant tumors, such as breast and gastric cancers (38, 39). We hypothesized that hematogenous and lymphatic dissemination caused by metastatic sites may increase the occurrence of distant metastases. Further studies focusing on this topic are warranted.

In clinical practice, CT scanning is often used to detect lung metastases; however, the detection of early metastatic lesions in the lung using this imaging technique is challenging (20). Conversely, 64-slice MDCT has great advantages for detecting small lung nodules (21). Patients poor tumor grade, squamous cell carcinoma histological type, T4 staging, late N staging, and more extrapulmonary metastatic sites, as well as black patients, should receive increased attention during clinical examination. Specifically, we suggest that these patients should undergo 64-slice MDCT scanning for lung nodules screening and, if necessary, pulmonary puncture pathology to ensure the early diagnosis of lung metastases. Timely diagnosis is important to assure that patients will receive appropriate treatment as soon as possible, significantly prolonging their survival and improving their quality of life (11, 17, 31).

In our study, we used competing risks analysis to identify variables affecting esophageal cancer-specific mortality. This analysis is based on events that occurred prior to the primary event of interest. Therefore, when predicting disease-specific outcomes, competing risk analysis provides a better estimation for the clinical prognosis of patients, helping clinicians to apply appropriate therapy strategies (40). Multivariable Cox regression analysis among patients with lung metastases indicated that patients with other histology types (e.g., signet ring cell carcinoma) had poorer OS than those with adenocarcinoma owing to their more aggressive biological behaviors (41). Interestingly, patients with T2-stage tumors had a better prognosis than those with T1-stage tumors. This finding may have been influenced by T staging inaccuracies in the SEER database.

Our study showed that patients who were 75 years old or older and with more extrapulmonary metastatic sites had a significantly lower overall survival and cancer-specific survival, while patients who received positive treatment (radiotherapy, chemotherapy, or chemoradiotherapy) had better prognoses. Patients aged ≥75 years had a shorter survival time than those aged 18–58 years. This discrepancy may be a result of poorer physical fitness and reduced natural life expectancy of older adults. Furthermore, from our findings, we observed that more extrapulmonary metastatic sites were consistently associated with poorer survival, which is in accordance with the results of other studies (17, 18). This trend is similar to that observed in other malignant tumors (38, 39). Therefore, a higher number of metastatic sites may indicate a poor prognosis in malignant diseases in general.

As evidenced by the Kaplan–Meier curves, radiotherapy, chemotherapy, and chemoradiotherapy treatment increased the median survival of patients with ECLM by 1, 6, and 6 months, respectively, compared with no treatment. The median survival of patients who received chemotherapy or chemoradiotherapy did not differ significantly (*P* = 0.366). Furthermore, patients who received radiotherapy had a better prognosis than patients who received no therapy (*P* < 0.01). However, similarly to the results of another SEER-based study (42), radiotherapy did not significantly affect cancer-specific mortality. In contrast, other studies have reached different conclusions (12, 43). Differently from these studies, we focused on patients with ECLM and their cancer-specific mortality, which may explain the result discrepancy. A prospective study suggested that chemoradiotherapy was superior to radiotherapy alone for treating patients with esophageal cancer (44). Another retrospective study showed that patients with stage IV B ESCC who underwent multimodality therapy, especially chemoradiotherapy, had significantly better survival than those who underwent single-modality therapy and supportive care (11). Similarly, we also found that chemoradiotherapy was superior to radiotherapy alone (*P* < 0.01). Furthermore, Wang et al. (45) reported that local therapy with radiation (median dose, 5,040 cGy) after initial palliative chemotherapy could achieve better local control and long-term survival in patients with stage IV B esophageal cancer. Guttmann et al. (46) demonstrated that chemotherapy combined with definitive dose radiotherapy (≥5,040 cGy) to the primary tumor could improve survival in patients with metastatic esophageal cancer compared with chemotherapy alone, whereas chemotherapy combined with palliative dose radiotherapy (<5,040 cGy) had a slightly worse prognosis than that of chemotherapy alone. Thus, radiation dose has a significant impact on the prognosis of patients with metastatic esophageal cancer. Because the SEER database did not provide radiation dose information, we were unable to evaluate this aspect. This lack of radiotherapy dose data may also explain why we observed no differences in prognosis between chemotherapy and chemoradiotherapy.

The NCCN guidelines recommend solely palliative and supportive care for patients with metastatic esophageal cancer (16). Previous studies also indicated that patients with stage IV esophageal cancer should not undergo esophagectomy (14, 15). In our study, among 713 patients with ECLM, only 22 underwent surgery for primary tumor treatment. Thus, the number patients in the surgery sub-cohort was insufficient to perform a survival analysis and assess the effect of surgery on patients’ outcomes. Other studies have encountered this same limitation (18, 47). Therefore, randomized controlled and multicenter trials are required to determine whether surgery is an effective treatment option for patients with M1 esophageal cancer.

Presently, treatment strategies for esophageal cancer with distant metastasis remain controversial. Several studies, as well as the NCCN guidelines, demonstrated that surgery was inappropriate for M1 esophageal cancer because of patients’ short life expectancy and attendant risks (14–16). Moreover, no large-scale prospective studies have demonstrated whether surgery has a beneficial effect on the prognosis of patients with M1 esophageal cancer. Conversely, many studies showed that multimodality therapy (chemoradiotherapy) provided better treatment results for these patients (11, 43–45). In our study, most patients received chemoradiotherapy, achieving a median survival of 7 months. Over the past 10 years, targeted therapy (e.g., trastuzumab) for treating HER2-positive advanced and metastatic esophageal cancer has received increased attention and achieved positive results (13, 48). Thus, non-surgical multimodality treatments may represent the most appropriate choices for treating M1 esophageal cancer, including ECLM.

## Limitation

This study has some limitations. First, we utilized information from the SEER database, which is derived from a retrospective study and may carry inherent biases. Second, we only considered patients who had lung metastasis at initial diagnosis because data regarding patients who developed lung metastases during their disease course was not included in the SEER database. Third, all statistical analyses were based on the population of the United States and may not represent the population of other countries or regions. Fourth, education level and median family income were determined at a county level instead of a patient level, which may have affected the results of the uni- and multivariate analysis conducted in this study. Fifth, the SEER database did not include information regarding recurrence rate and mortality after treatment, which may have affected the evaluation of treatment effects. Finally, the SEER database did not provide details regarding chemotherapy drugs or radiotherapy dose and target. The incomplete information may have affected grouping accuracy, as well as the results derived from these groupings.

## Conclusion

To the best of our knowledge, this population-based study was the first to analyze patients with ECLM at initial diagnosis. Our study provided information regarding the epidemiology of lung metastases in these patients. Considering the factors that may predict the occurrence of lung metastasis at diagnosis, high-risk patients should undergo a 64-slice MDCT examination for small lung nodules screening. According to our findings, chemotherapy or chemoradiotherapy may represent the most advantageous treatments for patients with ECLM. Therefore, our study may provide useful information to help physicians in early diagnosis and selection of appropriate treatment for patients with ECLM, ultimately improving the outcomes of these patients.

## Data Availability Statement

Publicly available datasets were analyzed in this study. This data can be found here: https://seer.cancer.gov/.

## Author Contributions

JG, SZ, HL, and LZ contributed to the conception and design of this research. JG, SZ, HL, and LZ performed the data acquisition and analysis. JG and SZ wrote the first draft of the manuscript. MH, TL, and JZ wrote sections of the manuscript. All authors contributed to the article and approved the submitted version.

## Conflict of Interest

The authors declare that the research was conducted in the absence of any commercial or financial relationships that could be construed as a potential conflict of interest.
