# Supplementary material for: Lung Metastases in Newly Diagnosed Esophageal Cancer: A Population-Based Study
Source: Front Oncol. 2021 Feb 25;11:603953. doi: 10.3389/fonc.2021.603953 (PMC7947855; doi:10.3389/fonc.2021.603953)
Supplement: Supplementary file 1 [file DataSheet_1.docx]

**Supplementary** **Table 1.** Univariable Logistic Regression for the Presence of Lung Metastases at Diagnosis of Esophagus Cancer.

| **Variable** | **Patients,No** | | **Among Entire Cohort** | |
| --- | --- | --- | --- | --- |
|  | **Patients (n=10965)** | **With Lung Metastases**  **(n=713)** | **OR (95%CI)** | ***P* Value** |
| Sex |  |  |  |  |
| Male | 8867 | 594 | 1 (reference) | NA |
| Female | 2098 | 119 | 0.837 (0.684-1.026) | 0.087 |
| Age at diagnosis, Y |  |  |  |  |
| 18-58 | 2830 | 205 | 1 (reference) | NA |
| 59-74 | 5737 | 374 | 0.893 (0.748-1.065) | 0.209 |
| ≥75 | 2398 | 134 | 0.758 (0.605-0.949) | 0.016 |
| Tumor location |  |  |  |  |
| Upper | 739 | 51 | 1 (reference) | NA |
| Middle | 1731 | 121 | 1.014 (0.650-1.084) | 0.937 |
| Lower | 7988 | 485 | 0.872 (0.647-1.176) | 0.001 |
| Overlapping | 507 | 56 | 1.675 (1.126-2.493) | 0.011 |
| Pathology grade |  |  |  |  |
| Grade I | 730 | 18 | 1 (reference) | NA |
| Grade II | 4670 | 284 | 2.561 (1.580-4.151) | ＜0.001 |
| Grade III/IV | 5565 | 411 | 3.154 (1.955-5.090) | ＜0.001 |
| Histology type |  |  |  |  |
| Adenocarcinoma | 7050 | 441 | 1 (reference) | NA |
| Squamous | 3087 | 226 | 1.184 (1.003-1.398) | 0.047 |
| Others^a^ | 828 | 46 | 0.882 (0.645-1.205) | 0.429 |
| T staging^b^ |  |  |  |  |
| T1 | 3071 | 254 | 1 (reference) | NA |
| T2 | 1435 | 31 | 0.245 (0.168-0.357) | ＜0.001 |
| T3 | 4984 | 185 | 0.428 (0.352-0.520) | ＜0.001 |
| T4 | 1475 | 243 | 2.188 (1.812-2.640) | ＜0.001 |
| N staging^b^ |  |  |  |  |
| N0 | 5107 | 175 | 1 (reference) | NA |
| N1 | 4704 | 413 | 2.713 (2.262-3.252) | ＜0.001 |
| N2 | 661 | 64 | 3.021 (2.241-4.073) | ＜0.001 |
| N3 | 493 | 61 | 3.980 (2.926-5.413) | ＜0.001 |
| Race |  |  |  |  |
| White | 9394 | 570 | 1 (reference) | NA |
| Black | 995 | 94 | 1.607 (1.278-2.020) | ＜0.001 |
| Others^c^ | 586 | 47 | 1.343 (0.985-1.831) | 0.026 |
| Unknown | 35 | 2 | 0.933 (0.223-3.900) | 0.925 |
| Insurance status |  |  |  |  |
| Insured | 10472 | 666 | 1 (reference) | NA |
| Uninsured | 335 | 36 | 1.733 (1.244-2.527) | 0.002 |
| Unknown | 158 | 11 | 1.102 (0.594-2.043) | 0.758 |
| Marital status |  |  |  |  |
| Married | 6171 | 364 | 1 (reference) | NA |
| Unmarried | 4267 | 313 | 1.263 (1.080-1.477) | 0.003 |
| Unknown | 527 | 36 | 1.170 (0.820-1.688) | 0.386 |
| High school education  (per 10% increase) | 10965 | 713 | 1.172 (0.969-1.195) | 0.172 |
| Median household income  (per 20000 increase) | 10965 | 713 | 0.962 (0.848-1.092) | 0.554 |
| Extrapulmonary metastatic sites to liver, bone, brain, and others No. | | |  |  |

| **variable** | **Patients,No** | | **Among Entire Cohort** | |
| --- | --- | --- | --- | --- |
|  | **Patients (n=10965)** | **With Lung Metastases**  **(n=713)** | **OR (95%CI)** | ***P* Value** |
| 0 | 9250 | 293 | 1 (reference) | NA |
| 1 | 1353 | 294 | 8.487 (7.132-10.099) | ＜0.001 |
| 2 | 316 | 98 | 13.742 (10.541-17.917) | ＜0.001 |
| 3 | 46 | 28 | 47.553 (26.008-86.947) | ＜0.001 |

CI: confidence interval, OR: odds ratio;

^a^including signet ring cell carcinoma,Mucinous carcinoma,etc;

^b^according to the eighth edition of the AJCC Cancer Staging manual;

^c^including Hispanic,Asian,etc.

**Supplementary** **Table 2.** Unitivariable Cox Regression for All Cause Mortality Among Esophageal Cancer Patients With Metastatic Disease

| **Variable** | **Patients, No.** | | **All-cause Mortality** | |
| --- | --- | --- | --- | --- |
|  | **Patients (n=10965)** | **With metastatic**  **Disease (n=2626)** | **Hazard Ratio (95%CI)** | ***P* Value** |
| Sex |  |  |  |  |
| Male | 8867 | 2224 | 1 (reference) | NA |
| Female | 2098 | 402 | 0.880 (0.783-0.990) | 0.033 |
| Age at diagnosis, Y | |  |  |  |
| 18-58 | 2830 | 835 | 1 (reference) | NA |
| 59-74 | 5737 | 1326 | 1.152 (1.048-1.265) | 0.003 |
| ≥75 | 2398 | 465 | 1.513 (1.339-1.710) | ＜0.001 |
| Tumor location |  |  |  |  |
| Upper | 739 | 112 | 1 (reference) | NA |
| Middle | 1731 | 340 | 0.929 (0.741-1.164) | 0.523 |
| Lower | 7988 | 2008 | 0.840 (0.687-1.027) | 0.089 |
| Overlapping | 507 | 166 | 1.033 (0.801-1.333) | 0.802 |
| Pathology grade |  |  |  |  |
| Grade I | 730 | 69 | 1 (reference) | NA |
| Grade II | 4670 | 917 | 0.910 (0.701-1.182) | 0.480 |
| Grade III/IV | 5565 | 1640 | 1.155 (0.894-1.492) | 0.272 |
| Histology type |  |  |  |  |
| Adenocarcinoma | 7050 | 1826 | 1 (reference) | NA |
| Squamous | 3087 | 568 | 1.239 (1.121-1.370) | ＜0.001 |
| Others | 828 | 232 | 1.386 (1.199-1.604) | ＜0.001 |
| T staging |  |  |  |  |
| T1 | 3071 | 846 | 1 (reference) | NA |
| T2 | 1435 | 188 | 0.623 (0.521-0.744) | ＜0.001 |
| T3 | 4984 | 866 | 0.702 (0.634-0.778) | ＜0.001 |
| T4 | 1475 | 726 | 1.107 (0.997-1.229) | 0.058 |
| N staging |  |  |  |  |
| N0 | 5107 | 584 | 1 (reference) | NA |
| N1 | 4704 | 1471 | 0.826 (0.746-0.915) | ＜0.001 |
| N2 | 661 | 340 | 0.747 (0.645-0.866) | ＜0.001 |
| N3 | 493 | 231 | 0.849 (0.717-1.006) | 0.059 |
| Extrapulmonary metastatic sites to liver, bone, brain, and others No. | | |  |  |
| 0 | 9250 | 911 | 1 (reference) | NA |
| 1 | 1353 | 1353 | 1.334 (1.219-1.461) | ＜0.001 |
| ≥2 | 362 | 362 | 1.823 (1.589-2.092) | ＜0.001 |
| Race |  |  |  |  |
| White | 9394 | 2239 | 1 (reference) | NA |
| Black | 995 | 235 | 1.245 (1.080-1.436) | 0.003 |
| Others | 586 | 142 | 0.947 (0.787-1.140) | 0.568 |
| Unknown | 35 | 10 | 0.861 (0.447-1.657) | 0.654 |
| Insurance status | |  |  |  |
| Insured | 10472 | 2482 | 1 (reference) | NA |
| Uninsured | 335 | 113 | 1.417 (1.159-1.732) | 0.001 |
| Unknown | 158 | 31 | 0.814 (0.544-1.218) | 0.317 |
| Marital status |  |  |  |  |
| Married | 6171 | 1472 | 1 (reference) | NA |
| Unmarried | 4267 | 1050 | 1.228 (1.127-1.337) | ＜0.001 |

| **Variable** | **Patients, No.** | | **All-cause Mortality** | |
| --- | --- | --- | --- | --- |
|  | **Patients (n=10965)** | **With metastatic**  **Disease (n=2626)** | **Hazard Ratio (95%CI)** | ***P* Value** |
| Unknown | 527 | 104 | 1.062 (0.857-1.316) | 0.581 |
| High school education  (per 10% increase) | 10965 | 2626 | 1.066 (1.006-1.130) | 0.029 |
| Median household income  (per 20000 increase) | 10965 | 2626 | 0.908 (0.870-0.948) | ＜0.001 |
| Treatment |  |  |  |  |
| No | 2265 | 508 | 1 (reference) | NA |
| Ra | 727 | 294 | 0.707 (0.610-0.819) | ＜0.001 |
| Che | 1303 | 854 | 0.251 (0.223-0.283) | ＜0.001 |
| Che+Ra | 6670 | 970 | 0.229 (0.204-0.257) | ＜0.001 |

CI: confidence interval;

^a^including signet ring cell carcinoma,Mucinous carcinoma,etc;

^b^according to the eighth edition of the AJCC Cancer Staging manual;

^c^including Hispanic,Asian,etc.

^d^including No:Without Radiotherapy or Chemotherapy RA:Radiotherapy,Che:Chemotherapy,Ra+Che:Radiotherapy plus Chemotherapy;

**Supplementary** **Table 3.** Multivariable Cox Regression for All Cause Mortality Among Esophageal Cancer Patients With Metastatic Disease

| **Variable** | **Patients, No.** | | **All-cause Mortality** | |
| --- | --- | --- | --- | --- |
|  | **Patients (n=10965)** | **With metastatic**  **Disease (n=2626)** | **Hazard Ratio (95%CI)** | ***P* Value** |
| Sex |  |  |  |  |
| Male | 8867 | 2224 | 1 (reference) | NA |
| Female | 2098 | 402 | 0.791 (0.701-0.894) | ＜0.001 |
| Age at diagnosis, Y | |  |  |  |
| 18-58 | 2830 | 835 | 1 (reference) | NA |
| 59-74 | 5737 | 1326 | 1.076 (0.977-1.184) | 0.137 |
| ≥75 | 2398 | 465 | 1.318 (1.157-1.500) | ＜0.001 |
| Tumor location |  |  |  |  |
| Upper | 739 | 112 | NA | NA |
| Middle | 1731 | 340 | NA | NA |
| Lower | 7988 | 2008 | NA | NA |
| Overlapping | 507 | 166 | NA | NA |
| Pathology grade |  |  |  |  |
| Grade I | 730 | 69 | NA | NA |
| Grade II | 4670 | 917 | NA | NA |
| Grade III/IV | 5565 | 1640 | NA | NA |
| Histology type |  |  |  |  |
| Adenocarcinoma | 7050 | 1826 | 1 (reference) | NA |
| Squamous | 3087 | 568 | 1.238 (1.101-1.392) | ＜0.001 |
| Others | 828 | 232 | 1.470 (1.269-1.704) | ＜0.001 |
| T staging |  |  |  |  |
| T1 | 3071 | 846 | 1 (reference) | NA |
| T2 | 1435 | 188 | 0.675 (0.564-0.809) | ＜0.001 |
| T3 | 4984 | 866 | 0.838 (0.751-0.935) | 0.002 |
| T4 | 1475 | 726 | 1.109 (0.996-1.234) | 0.060 |
| N staging |  |  |  |  |
| N0 | 5107 | 584 | 1 (reference) | NA |
| N1 | 4704 | 1471 | 1.015 (0.914-1.127) | 0.785 |
| N2 | 661 | 340 | 1.082 (0.928-1.262) | 0.313 |
| N3 | 493 | 231 | 1.122 (0.940-1.339) | 0.203 |
| Extrapulmonary metastatic sites to liver, bone, brain, and others No. | | |  |  |
| 0 | 9250 | 911 | 1 (reference) | NA |
| 1 | 1353 | 1353 | 1.347 (1.225-1.482) | ＜0.001 |
| ≥2 | 362 | 362 | 1.991 (1.728-2.294) | ＜0.001 |
| Race |  |  |  |  |
| White | 9394 | 2239 | 1(reference) | NA |
| Black | 995 | 235 | 1.002 (0.852-1.178) | 0.983 |
| Others | 586 | 142 | 1.006 (0.830-1.221) | 0.949 |
| Unknown | 35 | 10 | 0.725 (0.370-1.418) | 0.347 |
| Insurance status | |  |  |  |
| Insured | 10472 | 2482 | 1 (reference) | NA |
| Uninsured | 335 | 113 | 1.102 (0.897-1.354) | 0.356 |
| Unknown | 158 | 31 | 0.685 (0.452-1.036) | 0.073 |
| Marital status |  |  |  |  |
| Married | 6171 | 1472 | 1 (reference) | NA |
| Unmarried | 4267 | 1050 | 1.133 (1.035-1.239) | 0.007 |

| **Variable** | **Patients, No.** | | **All-cause Mortality** | |
| --- | --- | --- | --- | --- |
|  | **Patients (n=10965)** | **With metastatic**  **Disease (n=2626)** | **Hazard Ratio (95%CI)** | ***P* Value** |
| Unknown | 527 | 104 | 1.020 (0.822-1.267) | 0.857 |
| High school education  (per 10% increase) | 10965 | 2626 | 0.976 (0.916-1.041) | 0.460 |
| Median household income  (per 20000 increase) | 10965 | 2626 | 0.914 (0.871-0.959) | ＜0.001 |
| Treatment |  |  |  |  |
| No | 2265 | 508 | 1 (reference) | NA |
| Ra | 727 | 294 | 0.700 (0.602-0.814) | ＜0.001 |
| Che | 1303 | 854 | 0.262 (0.231-0.296) | ＜0.001 |
| Che+Ra | 6670 | 970 | 0.249 (0.220-0.282) | ＜0.001 |

CI: confidence interval;

^a^including signet ring cell carcinoma,Mucinous carcinoma,etc;

^b^according to the eighth edition of the AJCC Cancer Staging manual;

^c^including Hispanic,Asian,etc.

^d^including No:Without Radiotherapy or Chemotherapy RA:Radiotherapy,Che:Chemotherapy,Ra+Che:Radiotherapy plus Chemotherapy;

**Supplementary** **Table 4.** Univariable Cox Regression for All Cause Mortality and Esophageal Cancer Specific Mortality Among Patients With Lung Metastases

| **Variable** | **Patients, No.** | | **All-cause Mortality** | | **Cancer-specific mortality** | |
| --- | --- | --- | --- | --- | --- | --- |
|  | **Patients (n=10965)** | **With Lung Metastases**  **(n=713)** | **Hazard Ratio (95%CI)** | ***P* Value** | **Hazard Ratio (95%CI)** | ***P* Value** |
| Sex |  |  |  |  |  |  |
| Male | 8867 | 594 | 1 (reference) | NA | 1 (reference) | NA |
| Female | 2098 | 119 | 0.958 (0.778-1.181) | 0.690 | 1.53 (0.967-2.43) | 0.069 |
| Age at diagnosis, Y | |  |  |  |  |  |
| 18-58 | 2830 | 205 | 1 (reference) | NA | 1 (reference) | NA |
| 59-74 | 5737 | 374 | 1.156 (0.965-1.385) | 0.115 | 1.50 (0.862-2.62) | 0.150 |
| ≥75 | 2398 | 134 | 1.446 (1.151-1.817) | 0.002 | 3.05 (1.713-5.43) | ＜0.001 |
| Tumor location |  |  |  |  |  |  |
| Upper | 739 | 51 | 1 (reference) | NA | 1 (reference) | NA |
| Middle | 1731 | 121 | 1.100 (0.782-1.547) | 0.583 | 0.98 (0.386-2.48) | 0.97 |
| Lower | 7988 | 485 | 1.072 (0.793-1.448) | 0.653 | 1.01 (0.446-2.30) | 0.98 |
| Overlapping | 507 | 56 | 1.501 (1.008-2.234) | 0.045 | 2.07 (0.821-5.24) | 0.12 |
| Pathology grade |  |  |  |  |  |  |
| GradeⅠ | 730 | 18 | 1 (reference) | NA | 1 (reference) | NA |
| Grade Ⅱ | 4670 | 284 | 0.995 (0.609-1.628) | 0.985 | 1.12 (0.285-4.43) | 0.87 |
| Grade III/IV | 5565 | 411 | 1.275 (0.784-2.074) | 0.327 | 1.21 (0.310-4.70) | 0.78 |
| Histology type |  |  |  |  |  |  |
| Adenocarcinoma | 7050 | 441 | 1 (reference) | NA | 1 (reference) | NA |
| Squamous | 3087 | 226 | 1.148 (0.971-1.358) | 0.105 | 1.24 (0.806-1.90) | 0.330 |
| Others^a^ | 828 | 46 | 2.007 (1.455-2.769) | ＜0.001 | 2.10 (1.120-3.94) | 0.021 |
| T staging^b^ |  |  |  |  |  |  |
| T1 | 3071 | 254 | 1 (reference) | NA | 1 (reference) | NA |
| T2 | 1435 | 31 | 0.566 (0.366-0.877) | 0.011 | 0.450 (0.112-1.81) | 0.26 |
| T3 | 4984 | 185 | 0.689 (0.562-0.844) | ＜0.001 | 0.667 (0.386-1.15) | 0.14 |
| T4 | 1475 | 243 | 1.136 (0.947-1.363) | 0.168 | 0.968 (0.623-1.50) | 0.89 |
| N staging^b^ |  |  |  |  |  |  |
| N0 | 5107 | 175 | 1 (reference) | NA | 1 (reference) | NA |
| N1 | 4704 | 413 | 0.873 (0.727-1.048) | 0.145 | 1.061 (0.664-1.70) | 0.81 |
| N2 | 661 | 64 | 0.827 (0.608-1.124) | 0.224 | 0.492 (0.173-1.40) | 0.18 |
| N3 | 493 | 61 | 0.938 (0.682-1.291) | 0.697 | 1.209 (0.576-2.54) | 0.62 |
| Extrapulmonary metastatic sites to liver, bone, brain, and others No. | | |  |  |  |  |
| 0 | 9250 | 293 | 1 (reference) | NA | 1 (reference) | NA |
| 1 | 1353 | 294 | 1.326 (1.119-1.570) | 0.001 | 1.25 (1.00-1.55) | 0.049 |
| ≥2 | 362 | 126 | 1.536 (1.216-1.941) | ＜0.001 | 1.36 (1.02-1.81) | 0.036 |
| Race |  |  |  |  |  |  |
| White | 9394 | 570 | 1 (reference) | NA | 1 (reference) | NA |
| Black | 995 | 94 | 1.148 (0.915-1.442) | 0.234 | 1.01 (0.564-1.80) | 0.98 |
| Others^c^ | 586 | 47 | 0.988 (0.724-1.349) | 0.939 | 1.18 (0.566-2.47) | 0.66 |
| Unknown | 35 | 2 | 3.136 (0.78-12.605) | 0.107 | NA | NA |
| Insurance status |  |  |  |  |  |  |
| Insured | 10472 | 666 | 1 (reference) | NA | 1 (reference) | NA |
| Uninsured | 335 | 36 | 1.245 (0.877-1.769) | 0.220 | 1.38 (0.635-3.01) | 0.42 |
| Unknown | 158 | 11 | 0.953 (0.493-1.842) | 0.887 | NA | NA |
| Marital status |  |  |  |  |  |  |
| Married | 6171 | 364 | 1 (reference) | NA | 1 (reference) | NA |
| Unmarried | 4267 | 313 | 1.096 (0.936-1.285) | 0.258 | 0.809 (0.541-1.21) | 0.30 |

| **Variable** | **Patients, No.** | | **All-cause Mortality** | | **Cancer-specific mortality** | |
| --- | --- | --- | --- | --- | --- | --- |
|  | **Patients (n=10965)** | **With Lung Metastases**  **(n=713)** | **Hazard Ratio (95%CI)** | ***P* Value** | **Hazard Ratio (95%CI)** | ***P* Value** |
| Unknown | 527 | 36 | 0.789 (0.548-1.136) | 0.201 | 0.983 (0.407-2.380) | 0.97 |
| High school education  (per 10% increase) | 10965 | 713 | 1.060 (0.952-1.180) | 0.285 | 1.150 (0.889-1.490) | 0.28 |
| Median household income  (per 20000 increase) | 10965 | 713 | 0.914 (0.845-0.990) | 0.027 | 0.965 (0.790-1.180) | 0.72 |
| Treatment^d^ | |  |  |  |  |  |
| No | 2265 | 179 | 1 (reference) | NA | 1 (reference) | NA |
| Ra | 727 | 102 | 0.629 (0.490-0.806) | ＜0.001 | 0.987 (0.631-1.544) | 0.950 |
| Che | 1303 | 204 | 0.256 (0.206-0.317) | ＜0.001 | 0.181 (0.093-0.354) | ＜0.001 |
| Che+Ra | 6670 | 228 | 0.235 (0.190-0.291) | ＜0.001 | 0.192 (0.103-0.360) | ＜0.001 |

CI: confidence interval;

^a^including signet ring cell carcinoma,Mucinous carcinoma,etc;

^b^according to the eighth edition of the AJCC Cancer Staging manual;

^c^including Hispanic,Asian,etc.

^d^including No:Without Radiotherapy or Chemotherapy RA:Radiotherapy,Che:Chemotherapy,Ra+Che:Radiotherapy plus Chemotherapy;
